# Safety and effectiveness of oral medium to high dose blonanserin in patients with schizophrenia: subgroup analysis from a prospective, multicenter, post-marketing surveillance study in mainland China

**DOI:** 10.1186/s12991-023-00467-w

**Published:** 2023-10-06

**Authors:** Yuan Yang, Hongmin Ge, Xijin Wang, Xuejun Liu, Keqing Li, Gang Wang, Xiaodong Yang, Huaili Deng, Meijuan Sun, Ruiling Zhang, Jindong Chen, Duanfang Cai, Hong Sang, Xianglai Liu, Guilai Zhan, Guijun Zhao, Haiyun Li, Zhiyuan Xun

**Affiliations:** 1 Department of Psychiatry, Tianjin Anding Hospital, Hexi District, Tianjin, 300222 China; 2 Department of Psychiatry, The First Psychiatric Hospital of Harbin, Harbin, Heilongjiang China; 3 Department of Psychiatry, Brain Hospital of Hunan Province, Changsha, Hunan China; 4 Department of Psychiatry, Hebei Provincial Mental Health Center, Baoding, Hebei China; 5 The National Clinical Research Center for Mental Disorders & Beijing Key Laboratory of Mental Disorders, Beijing Anding Hospital, Capital Medical University, Beijing, China; 6 Advanced Innovation Center for Human Brain Protection, Capital Medical University, Beijing, China; 7 Department of Psychiatry, Shandong Mental Health Center, Jinan, Shandong China; 8 Department of Psychology, Psychiatric Hospital of Taiyuan City, Taiyuan, Shanxi China; 9 Department of Pharmacy, Daqing Third Hospital, Daqing, Heilongjiang China; 10 Department of Psychiatry, Henan Mental Hospital, Xinxiang, Henan China; 11 Department of Psychiatry, and National Clinical Research Center for Mental Disorders, The Second Xiangya Hospital of Central South University, Changsha, Hunan China; 12 Department of Psychiatry, The Fifth People’s Hospital of Zigong, Zigong, Sichuan China; 13 Mental Health Center, Changchun Sixth Hospital, Changchun, Jilin China; 14 Institute of Mental Health, Hainan Provincial Anning Hospital, Haikou, Hainan China; 15 Department of Psychiatry, Xuhui Mental Health Center, Shanghai, China; 16 Department of Psychiatry, Guangyuan Mental Health Center, Guangyuan, Sichuan China; 17 Medical Affairs, Sumitomo Pharma (Suzhou) Co., Ltd. Shanghai, Shanghai, China

**Keywords:** Blonanserin, Medium to high doses, Safety, Effectiveness, Schizophrenia

## Abstract

**Background:**

Blonanserin (BNS) had been undergoing post-market surveillance (PMS) since September 2018. Using the surveillance data, we did this analysis to assess the safety and effectiveness of different doses of BNS to explore a sufficient dose range of BNS in Chinese patients with schizophrenia (SZ).

**Methods:**

A 12-week, prospective, observational, single-arm, multicenter, open-label PMS was conducted. In this analysis, we divided the patients from PMS into low, medium to high, and high dose groups based on the dose of BNS they received, with medium to high dose group being the focus. The Brief Psychiatric Rating Scale (BPRS) scores at week 2 or 4, 6 or 8, and 12 were calculated to evaluate the effectiveness of BNS in improving psychiatric symptoms. The safety of BNS was reported as the incidence of adverse drug reactions.

**Results:**

364 patients were included in the medium to high dose group, of which 321 completed the surveillance, with a dropout rate of 11.8%. The mean daily dose was 15.1 ± 1.92 mg. The BPRS total score was 50.1 ± 11.95 at baseline and decreased to 26.6 ± 7.43 at 12 weeks (*P* < 0.001). When compared with other groups, the median to high dose group achieved significantly more reduction in BPRS score at week 12 (*P* = 0.004 versus low dose and *P* = 0.033 versus higher dose). Extrapyramidal symptoms [EPS (46.4%)] were the most common adverse reactions in the medium to high group. The average weight gain during the surveillance was 0.5 ± 2.56 kg and prolactin elevation occurred in 2.2% patients. Most adverse reactions were mild.

**Conclusions:**

BNS at medium to high doses (mean 15.1 mg/d) significantly improved symptoms of SZ and was well-tolerated. Most ADRs were mild, and the likelihood of causing metabolic side effects and prolactin elevations was low. Medium to high dose of BNS is a more potent treatment choice for SZ.

*Trial registration number*: ChiCTR2100048734. Date of registration: 2021/07/15 (retrospectively registered).

**Supplementary Information:**

The online version contains supplementary material available at 10.1186/s12991-023-00467-w.

## Introduction

Schizophrenia (SZ) is a psychiatric disorder with a weighted lifetime prevalence about 0.6% in China [1]. Patients with SZ are prone to relapse, usually accompanied by repeated hospitalizations and persistent disability, and require long-term drug treatments [2]. Antipsychotic medications are mainstays in the treatment of SZ. It is widely recognized that the application of antipsychotic drugs at a sufficient dose and with adequate treatment episode duration is essential for the management of SZ. An adequate dose can help patients recover quickly from the acute stage of SZ, reduce the hospitalization rate, and improve the long-term prognosis [2, 3]. However, high doses of the drug may also increase the side effects of treatment. Previous studies had shown that higher doses of risperidone resulted in a higher blood concentration, and the total effective rate of the medium or high dose group was significantly higher than that of the low dose group [4]. Meanwhile, other studies revealed that EPS was very likely to occur when the oral dose of risperidone was ≥ 10 mg/d, which also affected the therapeutic effects [5]. A meta-analysis also indicated that higher dose of antipsychotics was superior to their minimum effective dose in the total and positive symptom scores in psychopathology, but inferior in terms of side effects including Parkinson's disease scores, diarrhea, akathisia, somnolence, and vomiting [6]. Therefore, it is critical to determine whether a high dose of a certain antipsychotic drug is not only more effective but also safe enough at the same time.

Blonanserin (BNS; trade name: Lonasen^®^) is a new atypical antipsychotic drug approved in China in 2017 for the treatment of SZ [7]. Clinical trial data showed that in addition to improving positive and negative SZ symptoms, BNS had a lower risk of weight gain, subthreshold change in glycated hemoglobin (HbA_1c_) [8], and hyperprolactinemia [9]. Although the incidence of extrapyramidal symptoms (EPS) was higher than some atypical antipsychotics, the overall safety and tolerance were good [10].

The effectiveness and safety of BNS at a mean dose of 12 mg/d for treating SZ patients has been demonstrated in a previous large real-world study [10], but the range of a more sufficient dose with tolerable safety and more treatment effect is still to be explored. In this report, we did a post hoc analysis of the surveillance study to explore a sufficient dose of BNS which will further help to optimize the treatment strategy.

## Participants and methods

### Participants

In this study, we included patients diagnosed with SZ and received BNS treatment. As this was a post-marketing surveillance (PMS) study, no other inclusion or exclusion criteria were set. And the diagnosis of patients was determined by investigators using International Classification of Diseases 10th Edition (ICD-10).

### Methods

Detailed methods were published in a previous article [10]. Briefly, this was a prospective, observational, single-arm, multicenter, open-label, post-marketing surveillance study conducted in 16 hospitals in mainland China from September 2018 to May 2020 (patients taking medium to high dose of BNS were from 15/16 sites) [10]. Eligible patients received BNS at an initial dose of 4 mg/dose twice daily with further dose increase and adjustment based on judgement of doctors (no more than 24 mg/d), and got follow-up visits at enrollment, 2 or 4 weeks, 6 or 8 weeks, and 12 weeks. In this article, we did a post hoc analysis by different dose groups to explore a sufficient dose range of BNS in Chinese SZ patients. The study protocols were approved by the ethics committees of the leading clinical site at the Second Xiangya Hospital of Central South University (ethics approval number 2018-093). With the exception of individual clinical sites that approved waivers, all patients signed the informed consent form. The authors assert that all procedures contributing to this work comply with the ethical standards of the relevant national and institutional committees on human experimentation and with the Helsinki Declaration of 1975, as revised in 2008.

### Assessments

#### Groups by different doses

Patients were divided into three groups based on the doses they received. According to the label and results of striatal dopamine D2 receptor occupancy by blonanserin [11, 12], low dose group was defined as patients who did not meet the criteria that BNS administration ≥ 16 mg/day lasting ≥ 2 weeks, and the remaining patients who took 24 mg/day BNS that lasted ≥ 2 weeks were defined as a higher dose group, those who did not meet the criteria of low dose or higher dose groups were defined as a medium to high dose group. As medium to high dose of BNS may be closer to a sufficient dose range than low dose and may have better tolerability than higher dose, this analysis would focus on the population receiving medium to high doses of BNS.

#### Effectiveness evaluation

The BPRS was used for effectiveness assessment. All the items in the scale use a 7-level scoring method ranging from 1 to 7 points. The criteria for each level are asymptomatic, suspicious or very mild, mild, moderate, moderately severe, severe, and extremely severe. A higher total score indicates a more severe disease, and a larger reduction in the total score represents a more significant effectiveness [13].

#### Safety evaluation

We reported the incidence of adverse drug reactions (ADRs) based on the judgment of investigators and coded using the Medical Dictionary for Regulatory Activities (MedDRA) developed by the International Council for Harmonisation (ICH).

#### Analysis set

For the entire surveillance study, safety was analyzed in the safety set (SS) defined as patients who had received at least one dose of BNS with the recordings of post-treatment safety evaluations, and effectiveness analysis was done in the full analysis set (FAS) including all the patients who had taken BNS at least once. This subgroup analysis also applied the above principles.

### Statistical methods

To analyze drug safety, we compared the incidence of various adverse events among dose-based groups, and the differences were tested for significance using the chi-squared test. For the comparison of body weight, analysis of variance (ANOVA) was used to compare the differences between groups, and an LSD *t*-test was used for pairwise comparisons between groups. Effectiveness analysis was done based on the comparison of the BPRS total score. ANOVA was used to compare the differences between groups, and an LSD *t*-test was used to perform pairwise comparisons between groups. Change of BPRS total score from baseline was further analyzed using a mixed-effects model for repeated measures (MMRM) with treatment group, visit time, and treatment group-by-visit time interaction as fixed effects, and the baseline BPRS total score as the covariate in FAS population. Two-sided *P* < 0.05 was defined as statistically significant. A paired t-test was used to compare values before and after medication at each visit point within the group. Statistical analyses were done by the use of SAS (Version 9.3 or above).

## Results

### Dose groups, baseline demographic and clinical characteristics

A total of 1018 patients were enrolled in the surveillance study, of whom 620 received low dose, 364 received medium to high dose, and 34 received higher dose of BNS. Of the 364 patients who were given medium to high doses of BNS, 321 (88.2%) participants completed the study. All the 364 patients were included in the safety and effectiveness analysis. The age range of the medium to high dose group was 11–72 years old, with an average age of 32.7 ± 13.19 years. The baseline demographic and clinical information of the subjects who completed the monitoring is shown in Table 1.

**Table 1:** Baseline general demographic and clinical characteristics of medium to high dose group

	Low dose group (*n* = 620)	Medium to high dose group (*n* = 364)	High dose group (*n* = 34)
Age, years			
< 18, *n* (%)	42 (6.8)	32 (8.8)	4 (11.8)
≥ 18 and ≤ 40, *n* (%)	402 (64.8)	242 (66.5)	27 (79.4)
> 40, *n* (%)	176 (28.4)	90 (24.7)	3 (8.8)
Gender			
Male, *n* (%)	245 (39.5)	141 (38.7)	11 (32.4)
Female, *n* (%)	375 (60.5)	223 (61.3)	23 (67.6%)
Nationality			
Han, *n* (%)	611 (98.5)	361 (99.2)	34 (100.0)
Others, *n* (%)	9 (1.5)	3 (0.8)	0 (0.0)
Weight (kg)			
Mean ± SD	65.97 ± 14.158	65.19 ± 14.037	69.17 ± 15.444
Anamnesis			
Yes, *n* (%)	286 (46.1)	46 (12.6)	5 (14.7)
No, *n* (%)	334 (53.9)	318 (87.4)	29 (85.3)
Medication history			
Yes, *n* (%)	56 (9.0)	193 (53.0)	26 (76.5)
No, *n* (%)	564 (91.0)	171 (47.0)	8 (23.5)

*SD* standard deviation

**Fig. 1:**
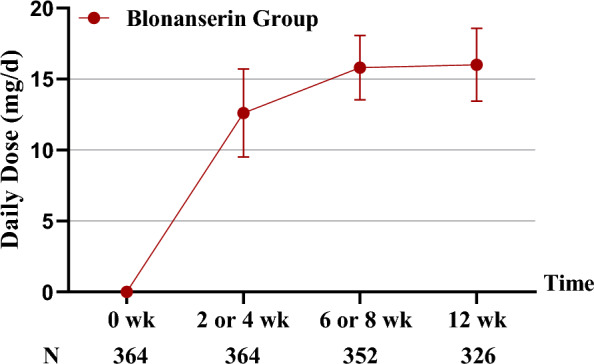
Daily dose of BNS in medium to high dose group over time (Mean ± SD)

The mean daily dose of BNS in the medium to high dose group during the surveillance was 15.1 ± 1.92 mg. The trend of mean daily dose over time is shown in Fig. 1.

### Effectiveness analysis in the medium to high dose group

The BPRS total score and each factor of the scale before treatment, at 2–4 weeks, 6–8 weeks, and 12 weeks of treatment (Fig. 1), are shown in Table 2. The results showed that the BPRS total score and the score of each factor at different visits were significantly different from the baseline. Compared to pre-treatment, LSD analysis showed that the BPRS total scores were significantly lower at 2–4 weeks (*P* < 0.001), 6–8 weeks (*P* < 0.001), and 12 weeks of treatment *P* < 0.001). For each item of the BPRS, treatment with the medium to high dose of BNS also achieved significantly better scores at all the time points compared with baseline (all *P* < 0.001, Table 2).

**Table 2:** BPRS total scores and scores of each factor for different treatment periods

Item	Before treatment (*n* = 364)	2–4 weeks of treatment (*n* = 362)	6–8 weeks of treatment (*n* = 349)	12 weeks of treatment (*n* = 322)
Anxiety-depression	10.0 ± 3.45	8.0 ± 2.75**	6.8 ± 2.49**	5.8 ± 1.96**
Anergia	10.2 ± 3.12	8.4 ± 2.55**	7.2 ± 2.39**	6.2 ± 2.03**
Thought disturbance	12.7 ± 4.08	9.5 ± 3.52**	7.7 ± 3.09**	6.3 ± 2.58**
Activation	6.6 ± 2.81	4.8 ± 1.97**	4.1 ± 1.50**	3.6 ± 1.21**
Hostility-suspiciousness	10.6 ± 3.78	7.3 ± 2.78**	5.8 ± 2.41**	4.7 ± 1.81**
BPRS total score	50.1 ± 11.95	38.0 ± 9.68**	31.5 ± 8.98**	26.6 ± 7.43**

*BPRS* brief psychiatric rating scale

**Compared to baseline, *P* < 0.001

In addition, this study also found that less patients dropped out in the medium to high dose group. At week 12, the dropout rate was 22.6% (140/620) in the low dose group, 11.8% (43/364) in the medium to high dose group, and 17.6% (6/34) in the higher dose group (see Additional file 1: Table S1).

The results of variance comparing different dose groups at different treatment times using an MMRM analysis are shown in Table 3. The reduction of BPRS total score was significantly larger in the medium to high dose group at 12 weeks, comparing with the low dose group. Regardless of the length of the treatment course, there was a statistical difference between the medium to high dose and the higher-dose groups; that is, more reduction in BPRS total score was observed in the medium to high dose group than the higher-dose group.

**Table 3:** Analysis (grouped according to dose) of change of BPRS total scores (before and after treatment)

Visit time	Mean	95%CIL	95%CIU	F	*P-*value
2 weeks or 4 weeks					
Low dose group	− 11.4	− 12.1	− 10.7		
Medium to high dose group	− 11.3	− 12.1	− 10.4		
Higher dose group	− 7.3	− 10.1	− 4.5		
Low vs. Medium to high	− 0.1	− 1.2	0.9	− 0.268	*0.789*
Low vs. Higher	− 4.1	− 7.0	− 1.2	− 2.810	*0.005*
Medium to high vs. Higher	− 4.0	− 6.9	− 1.0	− 2.663	*0.008*
6 weeks or 8 weeks					
Low dose group	− 16.6	− 17.3	− 15.9		
Medium to high dose group	− 17.6	− 18.5	− 16.7		
Higher dose group	− 12.6	− 15.6	− 9.6		
Low vs. Medium to high	1.0	− 0.2	2.1	1.682	*0.093*
Low vs. Higher	− 4.0	− 7.0	− 0.9	− 2.521	*0.012*
Medium to high vs. Higher	− 5.0	− 8.1	− 1.8	− 3.098	*0.002*
12 weeks					
Low dose group	− 20.0	− 20.8	− 19.3		
Medium to high dose group	− 21.8	− 22.8	− 20.8		
Higher dose group	− 18.2	− 21.4	− 15.0		
Low vs. Medium to high	1.8	0.6	3.0	2.876	*0.004*
Low vs. Higher	− 1.8	− 5.1	1.5	− 1.087	*0.277*
Medium to high vs. Higher	− 3.6	− 6.9	− 0.3	− 2.130	*0.033*

*BPRS* brief psychiatric rating scale, *CI* confidence interval

### ADR analysis in the BNS medium to high dose group

Mild ADRs occurred in 69 (19.0%) SZ patients treated with medium to high doses of BNS, and the proportions of moderate and severe ADRs were 31 (8.5%) and 3 (0.8%), respectively. The major ADRs of the nervous system included akathisia, tremor, dystonia, and Parkinsonism, mainly mild to moderate. Severe ADRs were rare (Table 4). Other ADRs in the nervous system are not shown in Table 4 because they were too infrequent or mild. Among all the abnormalities in various physical and biochemical examinations, mild ADRs were the most common in the medium to high dose group. The most frequent symptom was mild weight gain (2.7%), followed by a mild increase in blood prolactin (1.6%) (Table 4). No severe ADR was found in the hepatobiliary system, gastrointestinal system, and eye organs, as shown in Table 4. The occurrence of ADRs in other systems was not subdivided due to the low frequency.

**Table 4:** Most common ADR and ADR of interest in medium to high dose group

	Medium to high dose group (*N* = 364)					
	Mild		Moderate		Severe	
	Patients, *n* (%)	Events	Patients, *n* (%)	Events	Patients, *n* (%)	Events
Total^a^	69 (19.0)	205	31 (8.5)	86	3 (0.8)	7
Symptoms in the neuron system						
Akathisia	64 (17.6)	65	24 (6.6)	24	2 (0.5)	2
Tremor	43 (11.8)	45	12 (3.3)	12	0 (0)	0
Dystonia	13 (3.6)	13	12 (3.3)	13	1 (0.3)	1
Parkinsonism	12 (3.3)	13	8 (2.2)	8	1 (0.3)	1
Examines						
Weight gain	10 (2.7)	10	3 (0.8)	3	0 (0)	0
Hyperprolactin	6 (1.6)	6	1 (0.3)	1	1 (0.3)	1
Increased heart rate	1 (0.3)	1	4 (1.1)	4	0 (0)	0
Elevated transaminases	5 (1.4)	5	0 (0)	0	0 (0)	0
Elevated blood glucose	2 (0.5)	2	0 (0)	0	1 (0.3)	1
Hepatobiliary diseases						
Abnormal liver function	8 (2.2)	8	1 (0.3)	1	0 (0)	0
Gastrointestinal diseases	5 (1.4)	6	3 (0.8)	3	0 (0)	0
Eye diseases	4 (1.1)	6	1 (0.3)	1	0 (0)	0
Mental illness	1 (0.3)	1	2 (0.5)	2	0 (0)	0
Reproductive system and breast diseases	1 (0.3)	1	2 (0.5)	2	0 (0)	0
Metabolic and nutritional diseases						
Hyperlipidemia	2 (0.5)	2	0 (0)	0	0 (0)	0
Skin and subcutaneous tissue diseases	2 (0.5)	2	0 (0)	0	0 (0)	0
Systemic disease and administration site reactions	2 (0.5)	2	0 (0)	0	0 (0)	0

ADRs with a frequency of 1% or higher, or in the list of top 10, or of special interest were listed

*ADR* adverse drug reaction

^a^Categorized based on the severity of the most severe ADR encountered by each patient

To better describe the adverse reactions of patients taking BNS in different dose groups, we compared the data of the medium to high dose subgroup with those in the low dose group and the higher dose group obtained from the surveillance and found that the incidence of ADR varied among different dose groups. The number of patients that developed ADR was 103 in the medium to high dose group (*n* = 364, 28.3%), 91 in the low dose group (*n* = 620, 14.7%), and 11 in the higher dose group (*n* = 34, 32.4%). The P-value was < 0.001 for the overall comparison. Individually, *P* < 0.001 for the low dose group vs. medium to high dose group, *P* = 0.006 for the low dose group vs. higher dose group, and *P* = 0.617 for the medium to high dose group vs. higher dose group. The majority of ADRs were EPS in all dosage groups, of which the incidence rates were also significantly different (*P* < 0.001 for overall comparison; *P* < 0.001 for low dose group vs. medium to high dose group; *P* < 0.001 for low dose group vs. higher dose group).

## Discussion

This analysis focused on the exploration of the sufficient dose range of BNS, and its effectiveness and safety in SZ patients from the 12-week, prospective, observational, single-arm, open-label, multicentered post-marketing surveillance study. The medium to high dose of BNS had shown expected safety profile and was demonstrated as more effective in improving symptoms in SZ patients.

As the first surveillance report of medium to high doses of oral BNS in mainland China, this analysis demonstrated the improvement of patients with SZ treated with medium to high dose BNS. Previous study showed that striatal dopamine D2 receptor occupancy by blonanserin 16 mg/d was 73.4% (4.9%), which was within the range of 70% to 80% required for antipsychotic response [12]. And the average dose of BNS in the medium to high dose group during the surveillance turned to be 15.1 ± 1.92 mg/d. After 12 weeks of treatment, the symptoms of the SZ patients were effectively controlled within the group, reflected by the decrease of not only the total BPRS score but also the score of each factor, including anxiety-depression, anergia, thought disturbance, activation, and hostility-suspiciousness. Along with the prolonged treatment time, this suggested that BNS effectively improved both positive and negative symptoms of SZ patients, which is consistent with similar studies from other countries. For example, five post-marketing surveillance studies were carried out in Japanese patients with SZ and showed that the average total BPRS score at the last assessment was significantly lower than the baseline [13–17]. This is closely relevant to the pharmacological features of BNS. BNS has a potent inhibiting effect on the dopamine D2 receptor (Ki; 0.284 nM), dopamine D3 receptor (Ki; 0.277 nM), and serotonin 2A (5-HT2A) receptor (Ki; 0.640 nM) [17, 18]. The drug completely blocks the dopamine D2 and D3 receptors as well as serotonin 5-HT2A receptors but has low affinity to adrenergic alpha 1, serotonin 5-HT2C, histamine H1, and muscarinic M1 and M3 receptors, which is helpful to control symptoms and minimize ADRs [19].

When we further analyzed the difference in the change in BPRS total score between the medium to high dose group and the low dose group, we found that there was a statistical difference in score reduction between the two groups at the visit time of 12 weeks (*P* = 0.004). Meanwhile, at 2/4, 6/8, and 12 weeks after treatment, the score reduction in the medium to high dose group was more than that of the higher dose group, which indicated that the clinical effect was better in the medium to high dose group. Additionally, the drop-out rate was lowest in the medium to high dose group, implying that this dose group may better balance the effectiveness and safety and is a potentially better treatment choice. The treatment effect is related to the baseline characteristics of patients, such as the length of illness, whether they are treatment-naïve or not, and demographic characteristics. However, the above information was not completely collected in this study, and the influence of these factors on the treatment effect cannot be eliminated. Therefore, we still need further research. To achieve better efficacy, in addition to treating with an adequate dose, maintaining a certain treatment period is also required, which is in line with the principle of adequate dose and adequate time when treating SZ patients. Our analysis supports that the medium to high dose is closer to the “adequate dose”. In future studies, the adequate range of BNS for the treatment of Chinese SZ patients should be explored because different populations may have different metabolic dynamics of the drug. A review showed that race and ethnicity might lead to differences in drug exposure and/or response between individuals, which might alter the risk and benefit in certain populations [20]. About one-fifth of the new drugs approved in the past six years had shown differences in exposure and/or response across racial/ethnic groups, and the population-specific prescription was considered in very few cases [21]. Therefore, drug surveillance for patients in mainland China is of obvious significance because the prevalence of SZ in China has been increasing (the weighed lifetime and 12-month prevalence of SZ were both 0.6% in a survey conducted between 2013 and 2015) [1]. It is necessary and urgent to carry out detailed drug surveillance and to understand and optimize the clinical application of the drug, considering such a large patient population.

As medium to high dose of BNS might be more potent for treating SZ patients, it’s important to understand the safety of such treatment in the real-world setting. This analysis provided detailed side effects information according to each organ system and would serve as a valuable reference for the individualized use of BNS in clinical practice in China. This analysis showed that the most common adverse reactions when taking BNS at medium to high doses were EPS (46.4%), including akathisia (24.7%), tremor (15.1%), dystonia (7.1%), and Parkinsonism (5.8%). Elevated prolactin was also found in eight cases (2.2%). At the same time, the greatest weight gain was observed in the 12th week, with an average of 0.5 ± 2.56 kg. The above results are different from post-marketing surveillance data in Japan [13], in which the ADRs occurred in 730 (23.3%) of 3,130 patients, and the incidence of akathisia was the highest (4.3%), followed by hyperprolactinemia (2.8%), and EPS (2.4%). The proportion of concomitant medication in the medium–high dose group was 69.5% (253/364). And the post-marketing surveillance in China did not distinguish between patients with or without adjunctive antipsychotic treatment, which could cause a higher incidence of EPS. However, these differences are likely closely related to the different drug doses given to patients. Our analysis only included patients with daily doses of 16–24 mg/d of BNS, and no patients had a daily dose of more than 24 mg/d. Meanwhile, the proportion of patients in the Japanese study with a daily dose below 16 mg/d was as high as 78.6%, and the proportion of patients with a dose between 16 and 20 mg/d was only 3.1%. Thus, the adverse reactions increased along with the dose of BNS, and EPS was the most common, which is consistent with most other studies in China and other countries [9, 10, 14, 22]. Although the incidence of EPS (46.4%) in our analysis was numerically higher than that in Japan, it is still comparable with the results in similar studies. For example, the research conducted by Liu et al. [22], showed a 64.86% incidence of EPS. In the research, the BNS dose was 8–24 mg/d, and the average dose was 19.64 ± 4.36 mg/d in the 8th week of treatment, which was close to that of ours. In another study performed by Li et al., the incidence of EPS was 48.46% [9], which was even closer to our data; however, the relationship between dose and EPS could not be concluded because the average treatment dose was not reported. We found that the incidence of ADR in the medium to high dose group was higher than that of the low dose group, and the EPS incidence in the medium to high dose group was also higher than that in the low dose group. Therefore, the risk of EPS in the medium to high dose group was higher than that in the low dose group. However, cross-sectional comparison of the results was not possible due to the lack of studies with medium to high dose BNS. The relationship between hyperprolactinemia occurrence and dose of BNS is very close to the study in Japan. Our analysis supported that the increase in the treatment dose did not increase the incidence of hyperprolactinemia, which is also consistent with the conclusion of previous studies that “different doses of BNS did not cause a significant increase in blood prolactin levels” [23]. Similarly, no statistical significance was found in the incidence of weight gain when comparing the medium to high dose group with the low dose group. Therefore, we believe that BNS treatment with a medium to high dose has the most significant effect on the occurrence of EPS. However, most EPS events were mild to moderate which could be easily managed by doctors. In total, these results are of significance in furthering the understanding of physicians on the pharmacological characteristics of BNS.

There are several limitations to this analysis. First, this was conducted based only on the drug surveillance of BNS in the treatment of SZ patients without involving a control group using other antipsychotic drugs. Thus, a comparison of the efficacy with other antipsychotic drugs was not available. Second, samples were not collected for blood drug concentration analysis in this analysis. To further determine the optimal recommended dose for Chinese patients with SZ, it is necessary to design randomized controlled trials with the measurement of the blood drug concentration. Third, the patients were not further divided into subgroups. In Japan, surveillance-based studies had been conducted in patients with acute SZ, with diabetes [16], and with SZ as the primary diagnosed disease [14]. The difference in the use of BNS in different patient populations warrants further investigation in further studies. Finally, the cognitive function of patients with SZ was not evaluated, so the advantages of this drug in improving cognitive function in patients were not able to be assessed, which would also be another future research goal. Further investigations in the future could delve into comparative analyses with other antipsychotics. This could extend to comparisons among distinct disease courses or demographic characteristics, or patients with or without concomitant medications. Additionally, the inclusion of dedicated neurocognitive assessment scales to evaluate cognitive functioning is also a potential avenue for exploration.

## Conclusions

In summary, medium to high dose oral BNS can effectively improve various symptoms (including negative and positive symptoms) in patients with SZ, and it is also with acceptable safety profile, though some specific attention needs to be paid to the side effect of EPS. Medium to high dose of BNS (mean 15.1 mg/d) is a more potent treatment choice for SZ.

### Supplementary Information

**Additional file 1: Table S1.** Reasons for discontinuation.

## Data Availability

The datasets used and/or analyzed during the current study are available from the corresponding author on reasonable request.

## Abbreviations

BNS: Blonanserin
PMS: Post-market surveillance
SZ: Schizophrenia
BPRS: Brief Psychiatric Rating Scale
EPS: Extrapyramidal symptoms
ADRs: Adverse drug reactions
SS: Safety set
FAS: Full analysis set
ANOVA: Analysis of variance
MMRM: Mixed-effects model for repeated measures
