# Supplementary material for: Safety and effectiveness of oral medium to high dose blonanserin in patients with schizophrenia: subgroup analysis from a prospective, multicenter, post-marketing surveillance study in mainland China
Source: Ann Gen Psychiatry. 2023 Oct 6;22:37. doi: 10.1186/s12991-023-00467-w (PMC10557194; doi:10.1186/s12991-023-00467-w)
Supplement: Supplementary file 1 — Additional file 1: Table S1. Reasons for discontinuation. [file 12991_2023_467_MOESM1_ESM.docx]

**Safety and effectiveness of oral medium to high dose blonanserin in patients with schizophrenia: subgroup analysis from a prospective, multicenter, post-marketing surveillance study in mainland China**

**Table S1. Reasons for discontinuation**

|  | **Low dose group**  **(n=620) N (%)** | **Medium to high dose group (n=364) N (%)** | **High dose group**  **(n=34) N (%)** | **P-value^a^** |
| --- | --- | --- | --- | --- |
| Total | 140 (22.6) | 43 (11.8) | 6 (17.6) | 0.001 |
| Loss to follow-up (including withdrawal) | 84 (13.5) | 15 (4.1) | 3 (8.8) | Low vs. Medium to high: 0.001  Higher vs. Medium to high: 0.322 |
| Poor compliance | 28 (4.5) | 10 (2.7) | 2 (5.9) | Low vs. Higher: 0.501 |
| AE | 5 (0.8) | 4 (1.1) | / |  |
| Progressive disease | 4 (0.6) | 3 (0.8) | / |  |
| Death | / | 1 (0.3) | / |  |
| Others | 19 (3.1) | 10 (2.7) | 1 (2.9) |  |

a: Chi-squared test
